# Perceived climate change risk and global green activism among young people

**DOI:** 10.1007/s10260-023-00681-6

**Published:** 2023-01-30

**Authors:** Angela Maria D’Uggento, Alfonso Piscitelli, Nunziata Ribecco, Germana Scepi

**Affiliations:** 1 Department of Economics and Finance, University of Bari Aldo Moro, Largo Abbazia Santa Scolastica, 53, 70124 Bari, Italy; 2 Department of Agricultural Sciences, University of Naples Federico II, Naples, Italy; 3 Department of Economics and Finance, University of Bari Aldo Moro, Bari, Italy; 4 Department of Economics and Statistics, University of Naples Federico II, Naples, Italy

**Keywords:** Climate activism, Climate change mitigation, Environmental issues, Ordinal regression tree, Random forest

## Abstract

In recent years, the increasing number of natural disasters has raised concerns about the sustainability of our planet’s future. As young people comprise the generation that will suffer from the negative effects of climate change, they have become involved in a new climate activism that is also gaining interest in the public debate thanks to the Fridays for Future (FFF) movement. This paper analyses the results of a survey of 1,138 young people in a southern Italian region to explore their perceptions of the extent of environmental problems and their participation in protests of green movements such as the FFF. The statistical analyses perform an ordinal classification tree using an original impurity measure considering both the ordinal nature of the response variable and the heterogeneity of its ordered categories. The results show that respondents are concerned about the threat of climate change and participate in the FFF to claim their right to a healthier planet and encourage people to adopt environmentally friendly practices in their lifestyles. Young people feel they are global citizens, connected through the Internet and social media, and show greater sensitivity to the planet’s environmental problems, so they are willing to take effective action to demand sustainable policies from decision-makers. When planning public policies that will affect future generations, it is important for policymakers to know the demands and opinions of key stakeholders, especially young people, in order to plan the most appropriate measures, such as climate change mitigation.

## Introduction

In recent years, people around the world are increasingly experiencing the effects of climate change in the form of extreme events such as devastating floods, storms, droughts and fires. While the long-standing problems of global warming and glacier melting were probably perceived as “far away” because the latter occurred mainly in remote places on the planet or the former originated in the atmosphere, the rapid acceleration of extreme events in industrialised countries has demonstrated the seriousness of the current environmental situation, exacerbated by the global energy, economic and food crisis (Fasanelli et al. 2020; Galli et al. 2019) and triggered by an unexpected conflict in Europe’s neighbouring countries. All these events may have served as triggers for climate activism and the collective mobilisation of citizens. More than adults, young people have raised the demand for more effective and urgent intervention by policy makers to protect the planet and have become protagonists of a global protest movement that has quickly captured the attention of public opinion. This movement is called Fridays for Future (FFF), after the day of the first global school strike in March 2019, which involved 1.6 million protesters worldwide (Wahlstrom et al. 2019, Martiskainen et al. 2020). Some months later, in September 2019, 7.6 million participants took part in the third global FFF Day of Protest for Climate Justice, and the strikes continue each year, leading de Moor et al. (2020, 2021) to call the FFF movement the largest globally coordinated climate protest in world history (de Moor et al. 2021, 2020). Youth are aware that they are the generation who will inhabit a planet that is already sick and whose situation is inevitably deteriorating. They protest against the negative externalities of economic development and believe that they are the main victims of the rise in temperature, water and air pollution caused by the unsustainable production of goods and consumerism responsible for the overconsumption and waste of natural resources. They are ready to fight for their right to a healthier and sustainable future and to make their voices heard to raise public awareness of this issue. They want to shake up those who still believe that environmental problems are far away from them, are not really addressed by them and therefore do not feel the need to incorporate ecological practises into their lifestyles (Rathzel and Uzzell 2009).

To investigate young people’s engagement with the dangerous effects of climate change, a survey was conducted in February 2020 among 1,138 high school students in Southern Italy who were interviewed via an online questionnaire. The research question was based on investigating awareness of the main environmental issues, their opinion on the environmental plans of local authorities and whether students were willing to make a paradigm shift based on concrete actions that can reduce or stop the waste of natural resources and the pollution of the planet. Based on these premises, they committed to sharing the goals of the FFF movement, actively participating in the strikes and making a positive contribution through everyday environmental practises. To perform the statistical analysis, we used a tree-based method since, in our opinion, it is easier to interpret. For example, using a classical model such as POM (Proportional Odds Model), we get as many coefficients as there are categories for both the response variable and the predictors, all of them minus 1. The latter, applied to our data, would return 57 regression coefficients for the additive model (main effects) only. In this case, interpretation is difficult and we find that the classification tree for ordinal responses is much more interpretable when a data set contains many categorical variables with many categories. In our analysis, we applied an ordinal classification tree with the original impurity measure proposed by Morrone et al. (2019). The novelty of the ordinal tree methodology used in this paper allows for better discrimination of paths when the response variable is ordinal with a cut-off value that separates ordinal categories with a positive semantic meaning from those with a negative one.

The results of the study could be of interest to policy makers to understand how young people see their future from a sustainable perspective and to what extent they are ready to support the ecological transformation of public services provided to citizens. At a time when European countries need to put into practice the strategic guidelines to achieve the Sustainable Development Goals SDGs of the UN agenda (United Nations 2015), this study could be a contribution that gives some suggestions to public administrators and policy makers. It is not trivial to mention that the Italian government has put almost 250 billion euros in the reconstruction plan after COVID-19, the so-called National Recovery Plan (PNNR), to carry out projects and initiatives that will influence the destiny of the country not only towards repairing the damage caused by the COVID-19 pandemic but, above all, to leave a more sustainable country for the next generation. Among the six missions of the PNNR, the green revolution, ecological transition and sustainable mobility infrastructures occupy an important place. Great attention will be paid to supporting young people and the south of Italy, traditionally poorer than the north, which will receive more than 50% of the infrastructure budget to reduce the mobility gap (Agenzia Nazionale Stampa Associata—ANSA 2021).

The paper is organized as follows: Sect. 2 reviews the literature. In Sect. 3, we illustrate the survey on the perception of environmental risks in young people and their means of reaction. In Sect. 4, we describe and formalise the statistical methods used for the data analysis. The main results are illustrated in Sect. 5. The paper ends by summing up the results and discussing our main remarks.

## Climate activism and mitigation strategies

Climate change activism has been discussed by scholars from different angles. Roser-Renouf et al. (2014) addressed the question of cognitive and affective underpinnings to understand the genesis of climate change activism. Kleres and Wettergren (2017), seeking to understand how core emotions influence activists' motivations and mobilisation strategies, showed that fear plays a key role in raising awareness of the dangerousness of climate catastrophes and that hope drives collective movements, sharing the theme theorised by Nairn (2019). O’Brien et al. (2018) explored youth activism on climate change by arguing about dutiful, disruptive and dangerous dissent, three different types of behaviours that can be adopted by young people, and, following Corner et al. (2015), they expressed concern that their personal engagement may decrease if young people perceive their self-efficacy as limited. Fisher and Nasrin (2021) debated on a specific form of activism called civic engagement, a form of activism aimed to pressure different kinds of actors who might address the issue of climate change by adopting different tactics. In particular, they argued a different level of commitment depending on actors: citizens participating to influence communities, politicians and businesses (through strikes) can directly engage in lifestyle change by modifying their individual behaviour and consumption patterns (e.g. driving and flying less, using renewable energy and eating less dairy or meat) (Büchs et al. 2015; Cherry 2006; Cronin et al. 2014; Haenfler et al. 2012; Middlemiss 2011; Salt and Layzell 1985; Saunders et al. 2014; Stuart et al. 2013; Wynes and Nicholas 2017; Wynes et al. 2018). There are still a few studies that look at the direct effects of participation in green activism movements on changes in resource consumption (Saunders et al. 2014; Vestergren et al. 2019, 2018). According to Fisher and Nasrin (2021), it is also important to distinguish between direct and indirect pathways to achieve positive impacts on climate change by putting pressure on policy makers and companies to take emission-reducing measures. The direct pathway can be chosen simply through the adoption of the above-mentioned ecological behaviours by individuals. The other way works at a higher level by asking government policies to take into account the suggestions of scientists, and this is for example the strategy followed by international environmental non-governmental organisations (Dietz et al. 2015; Frank et al. 2000; Grant and Vasi 2017; Grant et al. 2018; Longhofer and Jorgenson 2017; Pfrommer et al. 2019; Schofer and Hironaka 2005; Setzer and Vanhala 2019). Some scholars (Ayling and Gunningham 2017; Franta 2017; Grady-Benson and Sarathy 2016) have discussed the special roles that the economic sector and businesses can play. In this case, people’s civic engagement has declined in the form of shareholder activism, which focuses on dissatisfied investors who, as shareholders, have the power to put pressure on the company to move towards social responsibility and environmental corporate activities and performance (Bratton and Mccahery 2015; Gillan and Starks 2007). Companies give due consideration to the expectations of their shareholders to address these issues in their strategic social responsibility documents (Hadden and Jasny 2017; Hestres and Hopke 2019; Yildiz et al. 2015).

Specifically related to the issue of the impact of people's actions in addressing climate change, a growing body of literature (Mi et al. 2019; Koehrsen 2021) has addressed climate change mitigation strategies, the pathways of which can be expressed through three main climate approaches (Leifeld and Menichetti 2018). The first approach addresses conventional climate mitigation efforts that use decarbonisation technologies capable of reducing CO2 emissions, namely renewables, fuel switching, efficiency improvements, nuclear energy and carbon capture, storage and use (Bataille et al. 2018; Fawzy et al. 2020). The second direction addresses a newer set of technologies and methods that can be implemented to capture CO2 from the atmosphere, referred to as negative emission technologies. They are based, for example, on methods for removing pollutants, storing bioenergy, increasing the alkalinity of the oceans, sequestering carbon in the soil, facilitating deforestation and reforestation (Goglio et al. 2020, Lawrence et al. 2018; Palmer 2019). The last direction of mitigation strategies is perhaps the most specialised, as it deals with extremely advanced technologies whose goal is to lower temperatures without altering greenhouse gas concentrations in the atmosphere (stratospheric aerosol injection, ocean sky brightening, cirrus cloud thinning and other techniques). However, as Lawrence et al. (2018) affirmed, the latter techniques are still theoretical in nature and cannot currently be included in policy frameworks. In this framework, which focuses on mitigation strategies that lie in the possibility of people’s intervention, it is interesting to consider two levels of intervention: the collective level, as a city, and the individual level, as an individual citizen-activist. They comprise more than half of the world’s population and are consequently responsible for three-quarters of global energy consumption and greenhouse gases (Gouldson, 2016). Many urban climate policies have been adopted to address climate change. The most important are improving energy efficiency, reducing fossil energy consumption and finding appropriate low-carbon development routes for sustainable development. All public organisations, especially local and regional authorities, need to clearly communicate their plans for environmental protection to improve citizens’ commitment to collective and synergistic action. From our point of view, it is interesting to understand the extent to which young people are informed about the policies and actions of local authorities and whether they feel committed as part of the community or as individuals. Based on the aims of our study, this difference is not trivial, as they could belong to at least two main profiles of young citizens. The first considers the community as a kind of “shield” in which other citizens work to achieve environmental goals; the second firmly believes that his or her own actions have a strong positive impact and are a necessary seed for the spread of ecological behaviours for a more sustainable future of the planet.

The issue of young people’s climate activism has been increasingly discussed by scholars in recent years. De Moor et al. (2021) defined two recent movements, FFF and Extinction Rebellion (XR), as “new” forms of climate activism because they had the power to inject new energy into global climate politics. To study the phenomenon in depth, they compared these movements with previous climate campaigns and found that the participants had some elements in common, while the main difference was the use of a more politically “neutral” framing of climate change. Recent studies have focused on the FFF movement and have made interesting contributions to knowledge about the phenomenon, social base and strategic choices of European youth (della Porta and Portos 2021), the communicative power of youth activism (Eide and Kunelius 2021) and crossing crises (Bowman and Pickard 2021; Martiskainen 2020). In particular, della Porta and Portos (2021) addressed the background of protesters as a possible trigger for an active role, noting that their social composition is heterogeneous, as there is a cross-class coalition that forms the collective mobilisation against climate change. In particular, social background may influence their opinions, as demonstrators from the upper class are more likely to believe that governments and corporations are capable of solving environmental problems than activists from the working and middle classes. Eide and Kurnelius (2021) focused on the ability of FFF activists to build an identity based on scientific evidence that strengthens their authority among climate policy actors. They are defined as new ambassadors for climate action who use networked communication tools to link personal experiences and add value to climate science. We believe that this could help encourage young people to engage in genuinely pro-environmental behaviour, and it is in line with de Moor et al. (2021), who argued that many FFF demonstrators who turned out to be students (Eide and Kunelius 2021; de Moor et al. 2020) embodied the belief that the climate crisis can be solved by individuals taking responsibility, and called on policymakers to address global warming on the basis of some kind of intergenerational justice.

Taking into account the recent literature on the subject, and with the aim of further contributing to the knowledge of the phenomenon, the basic idea of this study is to understand to what extent the only desirable scenario for a sustainable future is that citizens, together with businesses and policy makers, are willing to adopt environmentally friendly practises, even if they are more costly from a purely economic point of view or more demanding in terms of social behaviour. This can be illustrated in particular by the aim of understanding whether young people are willing to participate in strikes represented here by the FFF movement to defend the planet and adopt green practices as a way of life. In this way we can identify and compare their claimed principles with actual daily engagement. In such a globalised society, young people are most concerned about climate change (Calculli et al. 2021) and know its consequences, regardless of where they will occur on the planet, because they are its future inhabitants.

## A survey of young people’s perceptions of environmental risks and their climate activism

To discover how young people perceive the main risks of climate change and whether they are taking action to address the vulnerable impacts in their future, a survey was conducted in February 2020 among students and teachers in Puglia, a region in Southern Italy.

Student participation consisted of completing an anonymous online questionnaire for which formal privacy consent was obtained. The respondents belonged to Apulian high schools participating in the National Project for a Scientific Degree in Statistics (PLS), sponsored year after year by the Ministry of Education and Research.

The schools participating in the PLS program signed an agreement with the Italian universities concerned with carrying out activities that promote the acquisition of scientific and statistical skills that are more in demand in the labour market. In particular, this work draws on the experience of the University of Bari, which is one of the 14 Italian universities currently participating in the PLS and has been doing so since the 2010–2011 academic year (Ribecco et al. 2019). The PLS targeted a large number of high school students participating in the 2019–2020 PLS Project for Statistics.

The respondents were surveyed using an online questionnaire containing 32 questions divided into four sections: I. Sociodemographic information 2. Knowledge and awareness of environmental issues 3. Perception of environmental risks due to climate change. 4. Environmental awareness and agreement with the principles of the FFF movement. The respondents were asked to express their responses by selecting a few options (yes; no and I do not know) or more frequently by rating their responses on a five-point Likert scale (Likert 1932), with 1 being the lowest, 5 being the highest, and 3 being neutral.

A total of 1,793 questionnaires were collected, but 395 records were deleted during the cleaning and preparation data phase because they were not completed or proved unreliable or the response variable was missing; 260 questionnaires completed by adults (teachers and relatives) were not considered for analysis because this group did not fulfil the aims of the study. The final data frame contained 1,138 records and a subgroup of variables of interest were selected from a larger number, considering the responses that most closely matched the characteristics of the region of residence's exposure to climate change impacts.

As shown in Table A1 (see the Appendix), the students surveyed were almost evenly distributed by gender (50.2% female and 49.8% male). Their mean age was 15.9 years (± 1.4 standard deviation). In the year in which the survey was conducted, they were representative in terms of age and gender, as the proportion of female Southern Italian students to the total number of female Southern Italian students was 48.3% and the corresponding proportion of male Southern Italian students was 51.6%. Overall, female and male Southern Italian students represented 26.1% and 26.2% of Italian high school students, respectively (Ministry of University and Research—MIUR, 2022).

Table A1 also shows the percentage distribution of the variables analysed in the decision tree. From the data collected, more than 89.6% of the respondents believe that FFF can be effective in combating the destruction of the planet and has achieved important results, such as a slight reduction in environmental problems (23.9%), a global resonance to environmental issues (28.2%), and a call to policy makers around the world to take concrete action for greater sustainability (32.8%). Although the respondents showed a high level of commitment to and support for the principles of the FFF movement, only five out of 100 played an active role in environmental associations. In addition, the respondents believe they are well informed about environmental issues and are concerned about the various risks posed by climate change (extreme temperatures, flooding, fire, drought, storm surges and coastal erosion, tornadoes, etc.). Although they do not believe they live in a geographic area exposed to the most extreme phenomena, they see their country as moderately to highly vulnerable to climate change and its associated risks, especially extreme temperatures.

Strong environmental awareness is demonstrated by the adoption of ecological behaviours such as recycling, reducing waste and plastic consumption, using organic products and using public transport, bicycles and electric scooters. Students participating in FFF strikes often show an even higher rate of adoption of these ecological best practices.

## Methodology

In line with the aims of the study, the preliminary exploratory analysis allowed us to identify the distributions of the variables of interest. We hypothesized that young people who participate in the strikes of the FFF movement because they see their future in danger consistently adopt ecological practices as a lifestyle. To understand the factors that influence participation in FFF movement strikes, regression-like techniques could be used, but parametric methods do not always produce the expected results. On the contrary, data mining techniques based on recursion, averaging, and randomization are able to discover hidden paths and build better predictive models. We have a set of 22 predictors that are ordinal and nominal variables, and the response variable is measured on an ordinal scale. To examine the factors that affect the climate activism, we used a tree-based method based on the classification and regression tree (CART) proposed by Brieman (1984) but modified for ordinal response variables.

### Classification tree in brief

A classification tree identifies the relationships between a response variable, ***Y***, and a set of predictor variables (***X***_***1***_,***X***_***2***_,…,***X***_***p***_). In particular, a classification tree is a binary segmentation procedure of the data matrix to generate many more informative sub-partitions. In our opinion the use of a tree-based method instead of a (generalised) linear model is more effective because Ordinary Least Squares—based regressions (with no interaction terms) return one type of best fit to the data, namely a straight-line combination of the independent variables in a higher-dimensional space.

Moreover, the classification tree approach is chosen among the most commonly used supervised machine learning algorithms apt to cope with a categorical target, as the flexibility and robustness it offers to analyse such kind of data, a strong tolerance to missing responses and the absence of strict constraints in terms of distributional assumptions about the data—along with the intrinsic capability of addressing in an easy way interaction, nonlinear effects, and causal priorities—coupled with the possibility of attaining a high degree of interpretability of the classification rules, makes it a very good candidate for an explorative approach to our data (Fasanelli et al. 2017; Iorio et al. 2015; Piscitelli and D’Uggento 2022). Tree-based methods are often used in data mining contexts with large datasets to study, such as social science surveys.

For an extensive introduction to tree-based methods, we refer to Breiman et al (1984) and Hastie et al. (2013).

### Tree‐based methods for ordinal response variables

To take into account the ordinal nature of the response variable, Piccarreta (2008) suggested adopting of a new impurity measure based on the dispersion for ordinal data defined by Gini (1955):

1 $$D = 2\mathop {\mathop {\sum F_{Y} \left( i \right)\left[ {1 - F_{Y} \left( i \right)} \right]}\limits_{i = 1} }\limits^{h - 1}$$

where *F*_*Y*_(*i*) = P(*Y* ≤ *i*).

The proposal was originally implemented in an R package (Core Team 2022) by Archer (2010) and was then revised and technically corrected by Galimberti et al. (2012) in a new freely available R package named *rpartScore*.

Nonetheless, the formulation in Eq. 1 does not fit the case of an ordinal variable such as, for example, satisfaction, because we believe that the scale used to measure it has an implicit crisp cut point, which separates ratings or scores expressed by people judging themselves as satisfied from ratings or scores referred to those who are unsatisfied.

In our specific case, the response variable spans over 5 ordered categories (coded from 1, Never, to 5, Every time), and in our opinion, an interviewee can be considered an active participant in FFF movement strikes if their score is higher than 3 (Occasionally or Sometimes). Therefore, the threshold equal to 3 is considered the cut-point that identifies an active participant in FFF movement strikes in our opinion.

To explain with an example how to choose a suitable impurity measure for the case at hand, let us consider the following Table 1, summarizing five hypothetical frequency distributions of this response variable:

**Table 1:** Artificial distributions used to evaluate the impurity function

	y i	1	2	3	4	5
D1		1/2	0	0	0	1/2
D2		1/2	1/2	0	0	0
D3		0	0	0	1/2	1/2
D4		0	0	1/2	1/2	0
D5		1/5	1/5	1/5	1/5	1/5

It is worth noting that neither calculating the normalised diversity index of Gini for the five artificial distributions in Table 1 D1 (0.625), D2 (0.625), D3 (0.625), D4 (0.625) and D5 (1.00)—nor adopting the ordinal Gini formulation by Piccarreta – D1 (2.00), D2 (0.50), D3 (0.50), D4 (0.50) and D5 (1.60)—results reflected in a good choice; both formulas fail to address the substantial difference that we would see reflected in the impurity function related to a peculiar case like D4 with respect to D2 or D3. Therefore, some of the authors proposed an ad hoc formulation of the impurity function (Morrone et al. 2019), based on weighting the differences among the ratings/scores on the two sides of the crisp threshold between an active participant in FFF movement strikes and a non-active participant, so that the impurity measure used leads to nodes where the separation between active participants in FFF movement strikes and not active ones is maximised when pursuing splits.

The formula for obtaining our modified Gini impurity is as follows:

2 $$HI = H_{{{\text{norm}}}} \times \left( {\mathop \sum \limits_{i = 2}^{h} \left| {w_{i} y_{i} - w_{{\left( {i - 1} \right)}} y_{{\left( {i - 1} \right)}} } \right|*{\text{sign}}\left( {f_{{y_{i} }} \cdot f_{{y_{i - 1} }} } \right)} \right)$$

where the weights $w_{i}$ are defined as –1 if $y_{i} \le y^{*}$ and 1 otherwise, and the sum is taken over the scores that have a non zero frequency only (as can be easily obtained by means of the signum function, being $sign\left( {f_{{y_{i} }} \cdot f_{{y_{i - 1} }} } \right) = 1$ *if* $f_{{y_{i} }} \cdot f_{{y_{i - 1} }} > 0$ and 0 if at least one of the involved frequencies is null) and $H_{norm}$ is the usual normalized Gini index: $\frac{{h \times \left( {1 - \mathop \sum \nolimits_{i = 1}^{h} f_{{y_{i} }}^{2} } \right)}}{{\left( {h - 1} \right)}}$.

This choice enables discriminating the relevant cases as D4, as the following values can show: by setting ${y}^{*}$= 3, our index applied to the distributions shown in Table 1, returns the values 3.750, 0.625, 0.625, 4.375 and 10.000 for D1, D2, D3, D4 and D5, respectively. This aptly targets the objective of penalizing potential splits where the frequencies peak around the threshold ${y}^{*}$, since these splits would produce children nodes in which active and non-active participants are mixed together.

For more details about the proprieties of the corrected Gini diversity index for ordinal categorical variables, we refer to Morrone et al. (2019).

### The Random Forest method

The Random Forest (RF) method is a widely used approach for classification and regression (Breiman 2001). In brief, RF is an iterative process that builds a set of classification or regression trees (Breiman et al. 1984) using bootstrap samples iteratively drawn from the original learning data set. Observations not used to construct a tree are termed out-of-bag observations for that tree. To reduce the correlation between the trees in the forest, each split in each tree was identified by using the best among a subset of predictors randomly chosen at that node.

RF was used to further harness the informative value in our data, by strengthening the identification of influent variables via resampling. Instead of resorting to the ensemble method for prediction — something we are not interested in at this stage — we exploit RF as a tool to rank variables based on their ability to predict the response which is assessed by variable importance measures (VIMs).

Given an error measure M (e.g., error rate or mean squared error), VIM is defined as:

3 $$VIM_{j}^{M} = \frac{1}{ntree}\sum\limits_{t = 1}^{ntree} {\left( {MP_{tj} - M_{tj} } \right)}$$

where *ntree* is the total number of trees in the forest. *MP*_*tj*_ denotes the error of the *t* tree when predicting all observations that are out-of-bag for tree *t* after randomly permuting the values of the *j-th* predictor variable. *M*_*tj*_ indicates the above-mentioned error of tree *t* before permuting the values of the *j-th* predictor variable.

The RF method has the same advantages: it is not parametric, since no specific distribution of the response variable is assumed and does not require any specification of the type of relationship (linear or nonlinear) between the response variable and the predictors. Moreover, it provides results for a more robust assessment of the importance of the variable compared to classical tree-based methods. We used RF only to conduct a variables importance study and not to obtain the minimum prediction error.

For a review of RF methodology, we refer to Breiman (2001) and Boulesteix et al. (2012).

## Data analysis and results

The ordinal classification tree was used to examine the relationship between the decision to participate in the FFF protest movement, caused by the perception of being at risk from climate change and several variables, such as: perception of being exposed to the main consequences of climate change (extreme temperatures, floods, wildfires, storm surges, tornadoes, drought, etc.), individual level of information, ecological practises carried out on a daily basis to respond to environmental degradation and, finally, commitment to the principles and achievements of the green FFF movement. Table 2 shows the 22 predictors that we believe influence young people's decision to take action to improve the environmental situation at risk.

**Table 2:** Dataset description: *response variable* and selected predictors

Acronym	Description	Nature	# of categories	
*FFFPRT*	*Participation in FFF movement strikes*	*Ordinal scale*	*5*	
TMPEXT	Perceived level of vulnerability to extreme temperatures as a result of climate change	Ordinal scale	5	
LEVINF	Level of information	Ordinal scale	5	
EXTPH	Presence of extreme phenomena in residential area	Nominal scale	3	
PEREXT	Experience of extreme phenomena	Nominal scale	2	
ITAEXP	Perception of Italy being exposed to climate change	Ordinal scale	5	
FLOEXP	Perception of being exposed to floods	Ordinal scale	5	
FIREXP	Perception of vulnerability to forest fires	Ordinal scale	5	
STOSU	Perception of vulnerability to storm surges/coastal erosion	Ordinal scale	5	
TORN	Perception of vulnerability to tornadoes	Ordinal scale	5	
DREXP	Perception of vulnerability to drought	Ordinal scale	5	
SAFRISK	Perception of safety related to environmental risks in the places where one usually spends time	Ordinal scale		5
RECYC	Recycling of plastic, glass, paper and organic waste	Nominal scale	2	
BIOPR	Purchase of biological products	Nominal scale	2	
WASRED	Reduction of waste (water, energy, food, etc.)	Nominal scale	2	
BMUSE	Use of biodegradable materials	Nominal scale	2	
PUBTR	Use of public transport	Nominal scale	2	
REUSE	Use of renewable energies	Nominal scale	2	
NOPLAS	Reduction of plastic consumption	Nominal scale	2	
ELVEH	Use of electric vehicles and bicycle	Nominal scale	2	
FFFCCC	Effectiveness of FFF in combating climate change	Nominal scale	3	
FFFRES	Results achieved by the FFF movement in relation to climate change	Nominal scale	4	
ENVASS	Registration with an environmental association	Nominal scale	2	

**Fig. 1:**
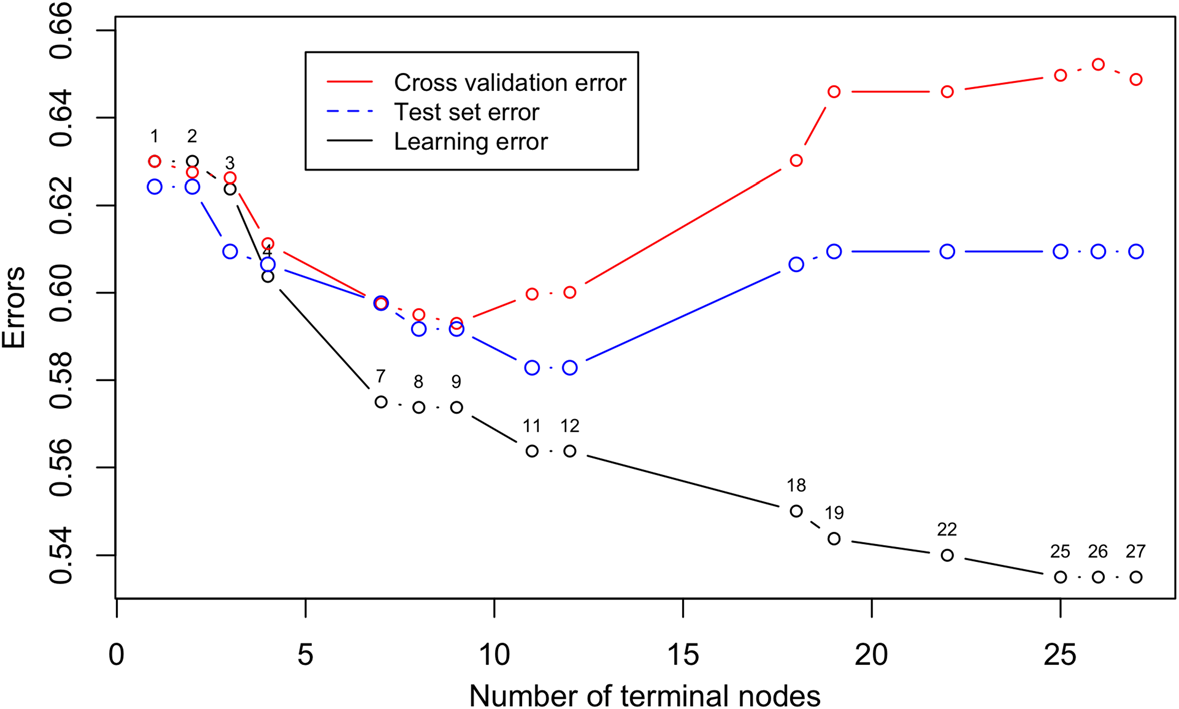
Decision tree selection

Data analysis was conducted with our own software written in R language (R Core 2022) on a computer with an Intel Core i7 quad-core processor (3.1 GHz). The dataset was randomly split into a “learning” set and a “test” set, with dimensions of 800 and 338 statistical units, respectively. The decision tree was selected via cross validation and the test set was used to estimate the prediction error in the tree pruning procedure (*cf*. Fig. 1).

**Fig. 2:**
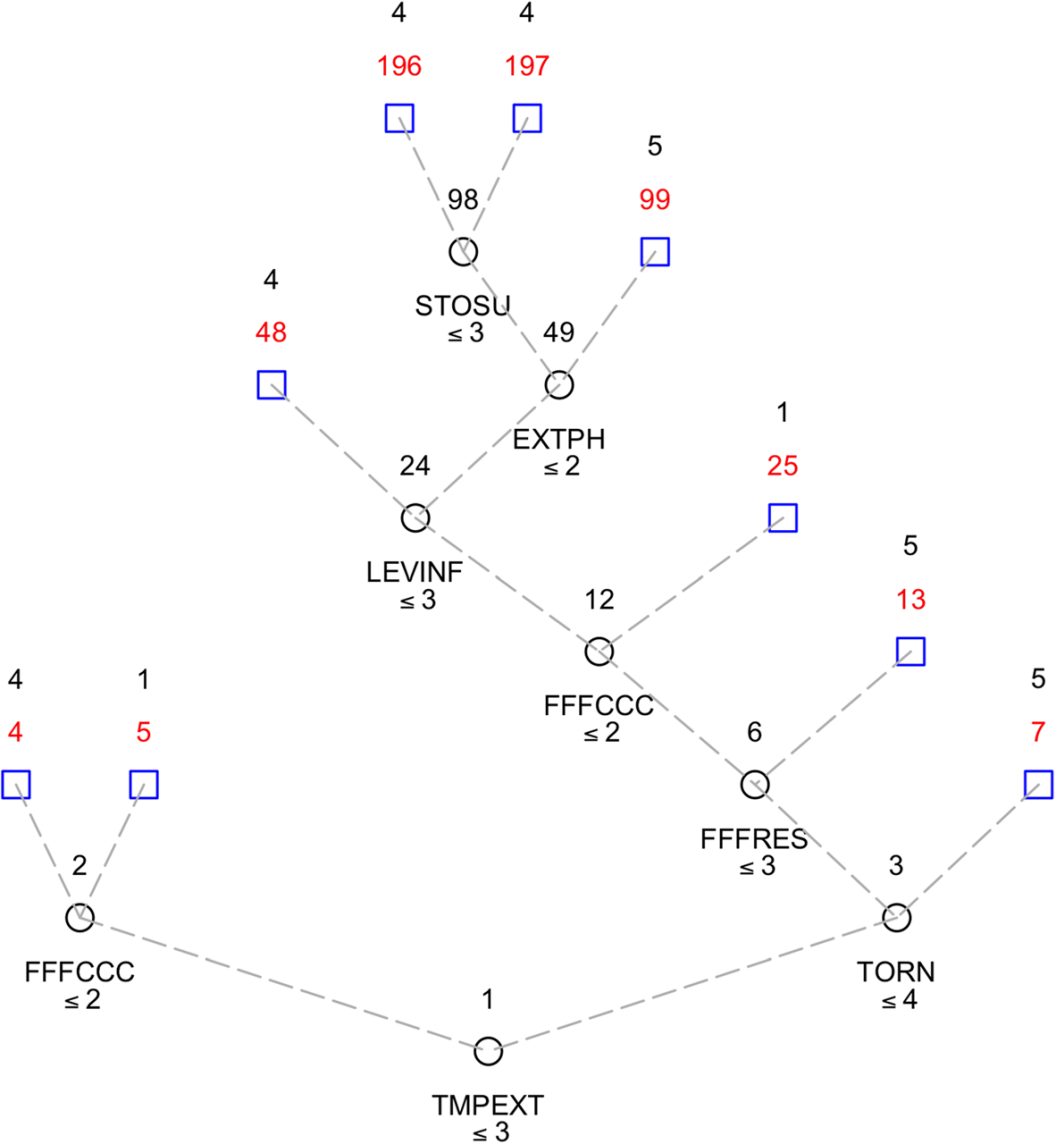
Ordinal regression tree

The minimum cross-validation error does not correspond to the minimum test set error, which would suggest a decision tree with 12 leaves. We chose the most conservative decision tree, which, in any case, confirms the interpretation of the phenomenon. The selected decision tree has L = 9 terminal nodes (Fig. 2). The cross-validation prediction error is equal to 0.5930 and the corresponding prediction error computed on the test set is equal to 0.5917. The tree graph allowed us to produce a partition of the sample of individuals into groups following the interactions between the predictors and the dependent variable (FFFPRT). Key information about each node is summarised in Table 3. The Perception of being exposed to extreme temperatures as one of the most frightening results of climate change is the first split (see Fig. 2) and separates the left side of the tree, with 51.1% of students who are from “not at all” to “somewhat” concerned about it, from the right one, where we find the students who considered themselves to be at a very high risk of experiencing extreme temperatures (from “moderately” to “extremely” dangerous, 48.9%).

**Table 3:** Classification tree in tabular form

Node	Size (prop)	Mode	Prop (1)	Prop (2)	Prop (3)	Prop (4)	Prop (5)	Path
5	49 (0.061)	1	0.429	0.204	0.265	0.102	0.000	TMPEXT ≤ 3 ∩ FFFCCC ∈ *No*
4	360 (0.450)	4	0.050	0.161	0.264	0.358	0.167	TMPEXT ≤ 3 ∩ FFFCCC ∈ *Yes*
7	31 (0.039)	5	0.000	0.000	0.000	0.419	0.581	TMPEXT > 3 ∩ TORN > 4
13	130 (0.163)	5	0.085	0.015	0.154	0.292	0.454	TMPEXT > 3 ∩ TORN ≤ 4 ∩
								FFFRES > 3
25	23 (0.029)	1	0.304	0.174	0.174	0.217	0.130	TMPEXT > 3 ∩ TORN ≤ 4 ∩
								FFFRES ≤ 3 ∩ FFFCCC ∈ *No*
48	114 (0.143)	4	0.000	0.061	0.193	0.535	0.211	TMPEXT > 3 ∩ TORN ≤ 4 ∩
								FFFRES ≤ 3 ∩ FFFCCC ∈ *Yes/better* ∩
								LEVINF ≤ 3
99	13 (0.016)	5	0.000	0.000	0.000	0.462	0.538	TMPEXT > 3 ∩ TORN ≤ 4 ∩
								FFFRES ≤ 3 ∩ FFFCCC∈ *Yes/better* ∩
								LEVINF > 3 ∩ EXTPH > 2
196	39 (0.049)	4	0.051	0.103	0.103	0.436	0.307	TMPEXT > 3 ∩TORN ≤ 4 ∩
								FFFRES ≤ 3 ∩ FFFCCC ∈ *Yes/better* ∩
								LEVINF > 3 ∩ EXTPH ≤ 2 ∩ STOSU ≤ 3
197	41 (0.051)	4	0.024	0.073	0.000	0.537	0.366	TMPEXT > 3 ∩ TORN ≤ 4 ∩
								FFFRES ≤ 3 ∩ FFFCCC ∈ *Yes/bettert* ∩
								LEVINF > 3 ∩ EXTPH ≤ 2 ∩ STOSU > 3

**Fig. 3:**
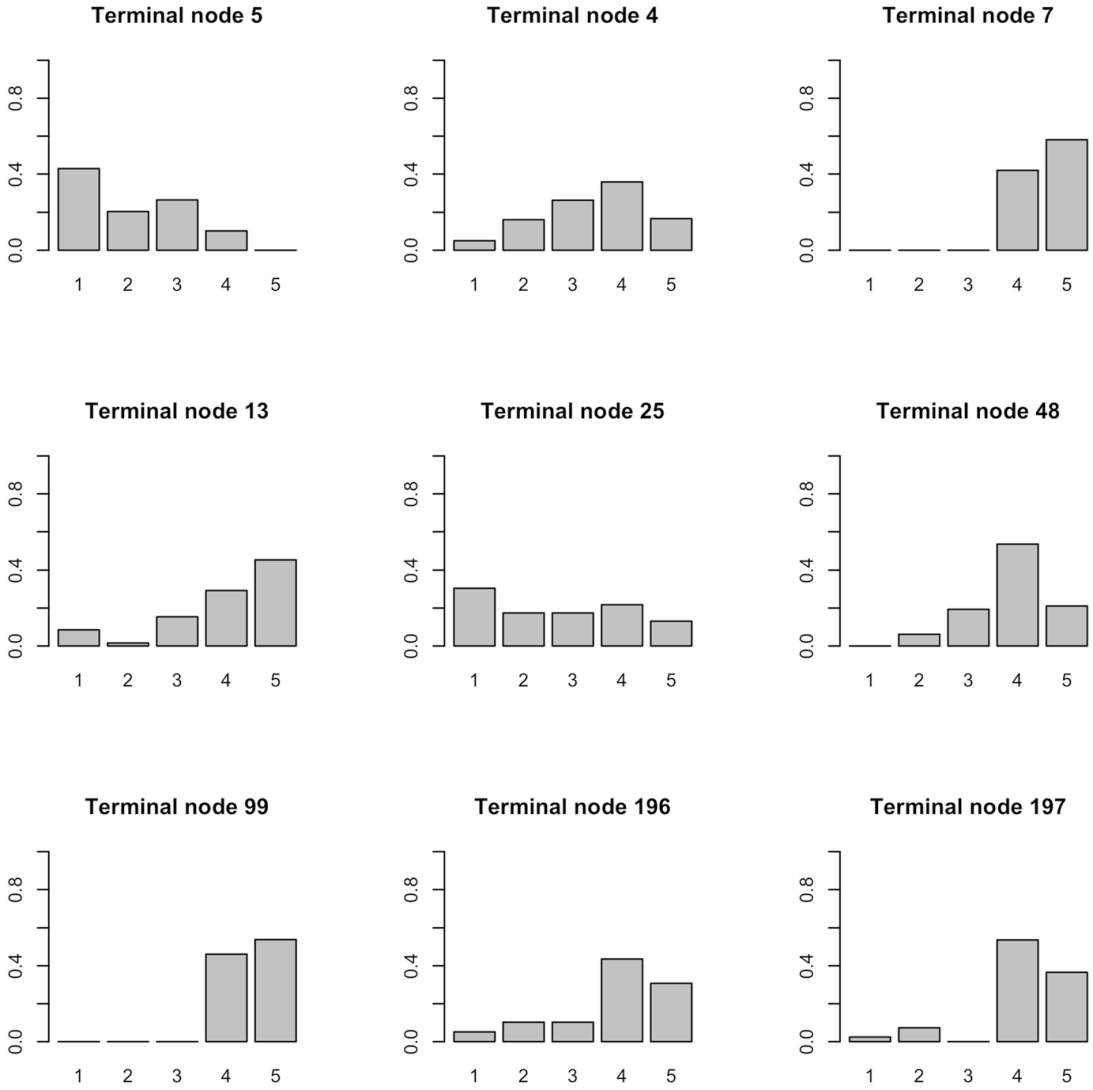
Distribution of the level of participation in FFF movement in the terminal nodes

The tree in Fig. 2 shows the role of predictors in the decision to protest by participating in the FFF movement (response variable) and to obtain the composition of the corresponding subgroups of student respondents. The tree has 9 terminal nodes and 8 splits corresponding to the following variables: TMPEXT, FFFCCC, TORN, FFFRES, LEVINF, EXTPH and STOSU. The information on the terminal nodes can be better assessed by examining the distribution of the variable FFF protest participation in each of them (see Table 3 and Fig. 3). Moreover, to obtain detailed information about the specific factors that trigger the FFFPRT response, it is interesting to analyse the pathways to the terminal nodes.

The first split is defined by the level of concern about experiencing extreme temperatures so that on the left side are students who have a medium to low concern and on the right are those who are highly scared.

Following the path in this branch, we find respondents who believe that participation in the movement is important (the modal values of the terminal nodes range from 3 to 5) and that the positive contribution of the FFF movement is to fight environmental degradation by raising people's awareness. A detailed analysis of terminal nodes number 4 and number 5 allows us to understand the combined effect of not being too scared about extreme temperatures exposition and to believe that FFF movement is able to produce effective results in contrasting climate change.

On the right side of the tree are respondents who are generally very concerned about the risk of being exposed not only to extreme temperatures, but also to other dangerous effects of climate change related to the physical and orographic characteristics of the area in which they live. This concern is closely related to an equally strong commitment to the results achieved by the green movement protest. In fact, the modal values of the response variables in the final nodes range between 4 and 5, indicating a high commitment to protest events in defence of the planet, as shown by all the terminal nodes created by the split at internal node number 3.

In particular, splitting at internal node number 6 separates students who perceive extreme temperatures, tornadoes, and storm surges as serious threats (terminal node 13), and who view the FFF’s main outcome as a call for policymakers to take concrete action on sustainability, from those who focus on the remaining options.

Returning to the main path leading to the top of the tree, other triggering factors seemed to additionally characterise the students interviewed. They strongly believe in the effectiveness of protest actions (split at internal node number 12) and have a medium level of information, but the split in node 24 highlights the respondents who consider themselves very well informed about environmental issues and see storm surges as the most likely feared impact of climate change occurring in their neighbourhood, which is not considered to be affected by extreme phenomena anyway.

The pathway leading to nodes 48, 99, 196 and 197 describes students who believe that the FFF is capable of drawing public attention to environmental problems and is thus an effective tool to reduce the destruction of the planet by raising people's awareness. This is consistent with the literature, as recent research reports that emotions such as fear and anger can trigger positive action on climate change (Kleres and Wettergren 2017; Martiskainen et al. 2020; Wang et al. 2018).

It is worth noting that the impact of potential risks emanating from the sea is felt more strongly than the others suggested (river floods, forest fires and drought), which is not surprising given that students live in close proximity to the Adriatic Sea.

Finally, an overview of the distribution of the level of engagement in the FFF protests in the 9 terminal nodes might help to better understand the paths just drawn (see Fig. 3). The paths leading to these distributions are shown in detail in the last column of Table 3.

The methodology of decision trees for ordinal variables provides terminal nodes that have the most powerful interrelations with the selected predictors. The paths to the terminal node are determined by the splitting criterion based on the impurity reduction approach of the CART algorithm. It allows us to look at the variable importance measures that led to the final solution.

**Fig. 4:**
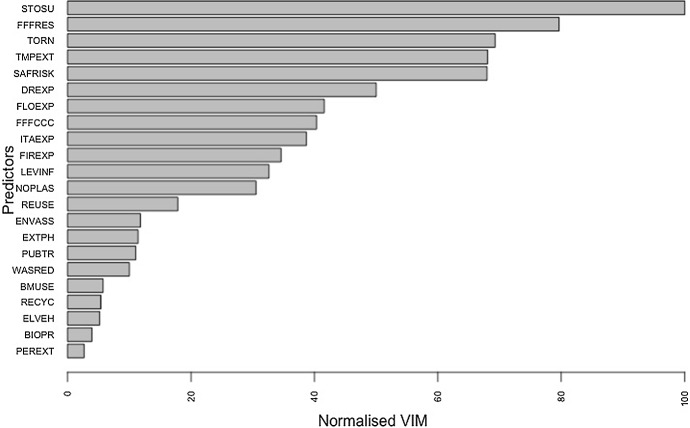
Predictors ordered according to their importance in the tree

Figure 4 shows the normalised importance plot for all predictor variables used in the tree. It confirms that extreme weather events, specifically storm surges, tornadoes, extreme temperatures, droughts and floods, have the same importance as the positive outcomes that can be achieved by people protesting to focus policy makers’ attention on sustainability.

It is clear that these predictors are more effective than the others which are at the bottom of the list and have decreasing importance, probably because they are mandatory behaviours and because they are seen as incapable of stopping environmental degradation at this time.

**Fig. 5:**
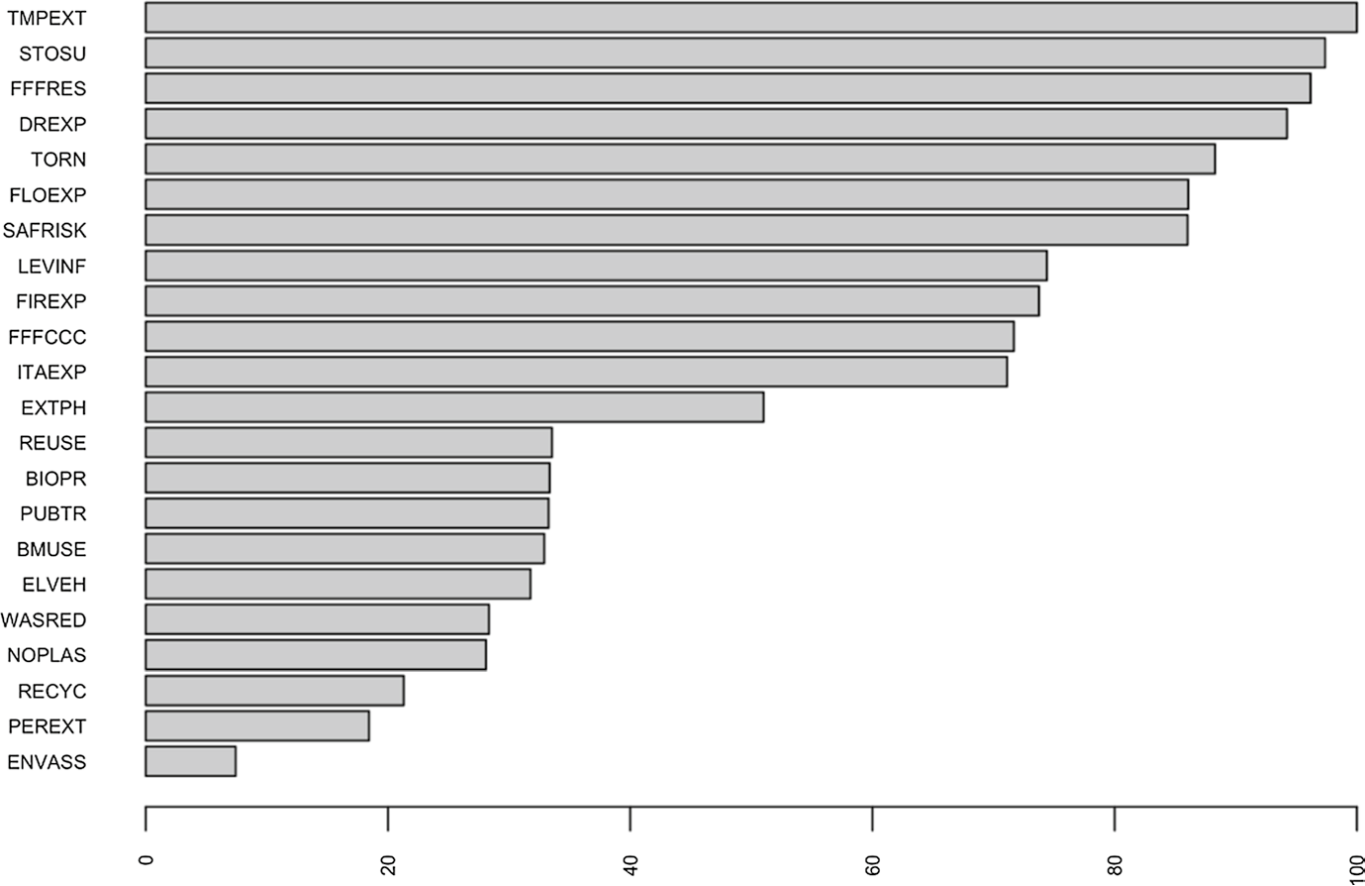
Predictors ordered according to their importance in RF

It is known that variable selection bias can occur with CART method, resulting in predictors with many cut-off points being selected without taking into account the information they provide (Hothorn et al. 2006; Loh and Shih 1997). To check for the presence of bias in our analysis, the RF technique was used (Brieman 2001). This technique was used to examine the importance of predictors in determining the levers that led the student respondents to protest and make their demands for a greener future in the interest of a global community. A forest of 5,000 trees was created to provide a robust ranking of predictors by importance. As can be seen in Fig. 5, the most important variable reducing overall uncertainty coincides with the first split, which relates to perceived levels of vulnerability to extreme temperatures due to climate change. Moreover, the most important predictors are the same as those shown in Fig. 4 in relation to the tree, albeit in different positions in the rank and this result confirms that the tree is robust. Overall, it is interesting to confirm that, among the ten main predictors in both analyses, six of them, namely STOSU, TORN, TMPEXT, DREXP, FLOEXP and FIREXP relate to the perception of vulnerability to natural disasters, the frequency of which has increased in recent years due to climate change.

The results that the FFF movement has achieved in raising awareness among public opinion, and especially among policymakers, about the dangerous consequences of the increasing destruction of the planet, as well as the belief in the effectiveness of FFF in combating climate change, play an important role in young people’s protests for a better future.

## Final remarks

Straining natural resources and the entire environmental system are increasing anthropogenic pressures on the Earth (Rockstorm et al. 2009) and are expected to have catastrophic consequences for humans if they are not stopped. Among the various approaches proposed by researchers to address the problem and find the best solution, Bengquist et al. (2019) focused on individuals changing their behaviour towards environmentally friendly behaviour to achieve a more sustainable future. A pro-environmental behaviour can be defined as one which “harms the environment as little as possible or even benefits the environment” (Steg and Vleck 2009, 309). Relatedly, a growing body of literature provides evidence that current mitigation efforts and future emissions commitments are unable to meet the temperature targets set out in the Paris Agreement (Lawrence et al. 2018); therefore, new ways of reducing emissions must be adopted. We share the theory of these scientists and hypothesise that, in theory, all measures taken by governments to protect the environment can benefit our planet in the long run, but many recent climate disasters have shown us that we have little time and we have to stop climate change by taking vivid actions. There is an urgent need for citizens and businesses to incorporate environmentally friendly practices into their lifestyles for the sake of a sustainable future.

Many people are aware of the seriousness of the environmental problem, but some probably still believe that it is happening in such a remote place that the negative consequences are not perceived as urgent (Rathzel and Uzzell 2009).

Since 2018, a global movement of students called FFF has drawn the attention of politicians and public opinion to the seriousness of the environmental situation, turning their fear of an uncertain future into a call for activism (Nairn 2019). FFF was able to mobilise many students who had experienced activism for the first time and felt the need to put pressure on politicians to listen to science (de Moor et al. 2021). The effectiveness of the FFF is more likely due to the fact that it is a global mobilisation wave composed of young people who are best informed about disasters and ecological risks through social media and traditional media, which contribute to increased concern. Many scholars (Wood 2020) believe that studies of young people's engagement with climate change are “a matter for closer investigation” about climate activism (de Moor et al. 2021) and provide a better understanding of the younger generation (Bowman and Pickard 2021). This study aims to contribute in this direction by examining students' perceptions of environmental issues and subsequent actions. We assume that young people who are so concerned about their future should take action, either by participating in school strikes or by acting ecologically in their everyday lives, to reduce or even stop the causes of climate change. To this end, approximately 1,100 high school students in a large city in Southern Italy were surveyed.

CART trees for ordinal data (Breiman et al. 1984; Galimberti et al. 2012; Morrone et al. 2019; Piccarreta 2008) were used to analyse the relationship between participation in the FFF movement and the willingness to adopt ecological practices as a lifestyle. Specifically, we used the CART method modified by introducing a new impurity measure for a distribution-free tree-based supervised classification method for ordinal response variables (Morrone et al. 2019). The proposed methodology is based on the assumption that the impurity measure must account for either the diversity or the order of the categories of the response variables. The novelty of the ordinal tree methodology proposed in this paper is its greater ability to distinguish groups based on the semantic value of the response categories. The classification system rewards individuals who give answers with the same semantics and distinguishes individuals with semantically opposite answers by using the central neutral answer as a cut-off. The results obtained in previous research (Morrone et al. 2019) confirm the suitability of the proposed approach (see, e.g., Diener et al. 1995 and the comments therein). In this study, both the response variable and the predictors are ordinal, and their categories exhibit diversity that must be handled correctly because they correspond to ranks that have the same absolute distance from each other but opposite semantic meaning on the left (negative) and right (positive) sides of the cut-off.

Through the responses collected, we were able to understand the levers that lead young people to act within the framework of climate activism.

It has been shown that the levers that drive students to protest and make their demands for a greener future in the interest of a global community are initially based on their concern about the negative impacts of climate change (natural disasters) and the awareness that the worsening situation will spiral out of control if we do not take immediate action. The responders are well informed about what is happening to the planet and view the environment as a whole system whose events can negatively affect their future. This awareness feeds into their environmentally friendly behaviours and lifestyles as they adopt key ecological practices, namely recycling plastic, glass, paper, and organic waste; using organic products and biodegradable materials; reducing waste (water, energy, food, etc.) and reducing plastic consumption. The students interviewed believed that global green movements such as FFF and climate activism in general could help effectively combat climate change by alerting policy makers to the urgency of environmental degradation.

## Appendix

See Table 4

**Table 4:** Main characteristics of respondents (*n* = 1,138)

Demographic characteristics	%	
Gender	Female	50.2
	Male	49.8
Age in year	Mean (standard deviation)	15.9 (± 1.4)
Classes (years)	13–15	38.0
	16–18	60.0
	> 19	2.0

Structural variables (ACRONYM) Categories	Category	%
Participation in FFF movements (FFFPRT)	1	4.7
	2	7.8
	3	31.3
	4	35.7
	5	20.4
Perceived level of vulnerability to extreme temperatures as a result of climate change(TMPEXT)	1	7.4
	2	16.3
	3	25.7
	4	28.9
	5	21.7
Effectiveness of FFF in combating climate change (FFFCCC)	No	10.4
	Yes but better	78.6
	Yes	11.0
Results achieved by the FFF movement in relation to climate change (FFFRES)	None	15.1
	Reduction	23.9
	Resonance	28.2
	Solicitation	32.8
Level of information (LEVINF)	1	1.5
	2	9.5
	3	46.2
	4	32.5
	5	10.3
Presence of extreme phenomena in residential area (EXTPH)	No	63.7
	I don’t know	16.0
	Yes	20.3
Experience of extreme phenomena (PEREXT)	Yes	14.8
	No	85.2
Perception of Italy being exposed to climate change (ITAEXP)	1	2.1
	2	8.8
	3	47.1
	4	32.4
	5	9.6
Perception of being exposed to floods (FLOEXP)	1	16.1
	2	29.5
	3	34.6
	4	15.6
	5	4.2
Perception of vulnerability to forest fires (FIREXP)	1	43.3
	2	31.1
	3	16.3
	4	6.7
	5	2.6
Perception of vulnerability to storm surges/coastal erosion (STOSU)	1	22.1
	2	24.3
	3	26.0
	4	19.8
	5	7.8
Perception of vulnerability to tornadoes (TORN)	1	22.1
	2	31.7
	3	25.3
	4	16.6
	5	4.4
Perception of vulnerability to drought (DREXP)	1	25.7
	2	27.0
	3	20.9
	4	17.4
	5	9.0
Perception of safety related to environmental risks in the places where one usually spends time (SAFERISK)	1	3.8
	2	14.0
	3	46.7
	4	27.5
	5	7.8
Recycling of plastic, glass, paper and organic waste (RECYC)	Yes	90.5
	No	9.5
	*% Yes in FFF*	*91.1*
Purchase of biological products (BIOPR)	Yes	40.1
	No	59.9
	*% Yes in FFF*	*42.1*
Reduction of waste (water, energy, food, etc.-WASRED)	Yes	74.6
	No	25.4
	*% Yes in FFF*	*76.7*
Use of biodegradable materials (BMUSE)	Yes	57.2
	No	42.8
	*% Yes in FFF*	*59.5*
Use of public transport (PUBTR)	Yes	63.1
	No	36.9
	*% Yes in FFF*	*68.4*
Use of renewable energies (REUSE)	Yes	51.7
	No	48.3
	*% Yes in FFF*	*50.1*
Reduction of plastic consumption (NOPLAS)	Yes	72.1
	No	27.9
	*% Yes in FFF*	*76.9*
Use of electric vehicles and bicycle (ELVEH)	Yes	42.6
	No	57.4
	*% Yes in FFF*	*42.3*
Registration with an environmental association (ENVASS)	Yes	5.1
	No	94.9
Knowledge about measures to combat climate change and manage risks taken by local authorities (KNENVMEA)	Yes	24.9
	I don’t know	33.5
	No	41.6
	*% Yes in FFF*	*29.8*
